# Plasma Metabolites Alert Patients With Chest Pain to Occurrence of Myocardial Infarction

**DOI:** 10.3389/fcvm.2021.652746

**Published:** 2021-04-23

**Authors:** Nan Aa, Ying Lu, Mengjie Yu, Heng Tang, Zhenyao Lu, Runbing Sun, Liansheng Wang, Chunjian Li, Zhijian Yang, Jiye Aa, Xiangqing Kong, Guangji Wang

**Affiliations:** ^1^Department of Cardiology, The First Affiliated Hospital of Nanjing Medical University, Nanjing, China; ^2^Department of Laboratory, The First Affiliated Hospital of Nanjing Medical University, Nanjing, China; ^3^Laboratory of Metabolomics, Jiangsu Key Laboratory of Drug Metabolism and Pharmacokinetics, China Pharmaceutical University, Nanjing, China

**Keywords:** myocardial infarction, risk factors, biomarker, metabolomics, arginine, deoxyuridine

## Abstract

Myocardial infarction (MI) is one of the leading causes of death worldwide, and knowing the early warning signs of MI is lifesaving. To expand our knowledge of MI, we analyzed plasma metabolites in MI and non-MI chest pain cases to identify markers for alerting about MI occurrence based on metabolomics. A total of 230 volunteers were recruited, consisting of 146 chest pain patients admitted with suspected MI (85 MIs and 61 non-MI chest pain cases) and 84 control individuals. Non-MI cardiac chest pain cases include unstable angina (UA), myocarditis, valvular heart diseases, etc. The blood samples of all suspected MI cases were collected not longer than 6 h since the onset of chest pain. Gas chromatography–mass spectrometry and liquid chromatography–mass spectrometry were applied to identify and quantify the plasma metabolites. Multivariate statistical analysis was utilized to analyze the data, and principal component analysis showed MI could be clearly distinguished from non-MI chest pain cases (including UA and other cases) in the scores plot of metabolomic data, better than that based on the data constructed with medical history and clinical biochemical parameters. Pathway analysis highlighted an upregulated methionine metabolism and downregulated arginine biosynthesis in MI cases. Receiver operating characteristic curve (ROC) and adjusted odds ratio (OR) were calculated to evaluate potential markers for the diagnosis and prediction ability of MI (MI vs. non-MI cases). Finally, gene expression profiles from the Gene Expression Omnibus (GEO) database were briefly discussed to study differential metabolites' connection with plasma transcriptomics. Deoxyuridine (dU), homoserine, and methionine scored highly in ROC analysis (AUC > 0.91), sensitivity (>80%), and specificity (>94%), and they were correlated to LDH and AST (*p* < 0.05). OR values suggested, after adjusting for gender, age, lipid levels, smoking, type II diabetes, and hypertension history, that high levels of dU of positive logOR = 3.01, methionine of logOR = 3.48, and homoserine of logOR = 1.61 and low levels of isopentenyl diphosphate (IDP) of negative logOR = −5.15, uracil of logOR = −2.38, and arginine of logOR = −0.82 were independent risk factors of MI. Our study highlighted that metabolites belonging to pyrimidine, methionine, and arginine metabolism are deeply influenced in MI plasma samples. dU, homoserine, and methionine are potential markers to recognize MI cases from other cardiac chest pain cases after the onset of chest pains. Individuals with high plasma abundance of dU, homoserine, or methionine have increased risk of MI, too.

## Introduction

A strangling feeling in the chest is a typical manifestation of coronary artery disease (CAD). In most cases, CAD develops as plaque builds up on the artery walls. When it progresses to myocardial infarction (MI), coronary heart disease will be life-threatening and extremely dangerous in the ensuing days or weeks due to its various fatal complications. Thus, its diagnosis and treatment is urgent. However, chest pain can be caused by other cardiovascular events or heart diseases (e.g., unstable angina, myocarditis) (1). Although there are cardiac damage biomarkers, such as creatine kinase-MB (CK-MB), aspartate aminotransferase (AST), and cardiac troponins (cTnT, cTnI), they are usually related to tissue damage only and not specific to MI. For example, cTnT elevation can also be observed in myocarditis, hypertrophic cardiomyopathy, sometimes unstable angina, etc. Sometimes, unexpected elevation of the markers can also be observed without obvious connection to cardiac injury (2–4).

With the development of metabolomics, more and more small molecule metabolic markers will be identified, analyzed, and studied. The newly found differential metabolites between MI cases and non-MI chest pain cases will expand our knowledge of myocardial infarction development. Since ischemic heart diseases are characterized by profound metabolic shifts at both the circulatory and local levels (5), metabolomics has been applied to study the metabolic pattern changes detected in the blood of CAD patients. Early in 2002, a pioneering work was published showing that NMR-based metabolomics had the potential to rapidly and non-invasively diagnose the presence and severity of coronary heart disease (6). In 2005, Marc Sabatine and his colleagues identified metabolic biomarkers of myocardial ischemia associated with physical exercise (7). Later studies focused on identifying biomarkers and metabolic pathways and exploring the underlying mechanisms associated with cardiovascular diseases (8–14). A panel of potential markers has been suggested for coronary heart diseases, such as arginine and homocysteine, and the underlying mechanisms of their action have been explored (15–17). However, candidate metabolites to recognize MI cases from other cardiac chest pain cases remain to be further studied and improved.

In this study, we mainly focus on detecting and assessing metabolites' ability to discriminate MI cases from non-MI chest pain cases. A metabolomic platform with GC/MS and LC/MS instrumentation was employed to profile plasma metabolites of hospitalized patients with chest pain (including MI and non-MI chest pain cases) and their controls. We first applied multivariate statistical analysis to address our question about how good these identified metabolites detect MI cases. Then, medical history and laboratory test variables (e.g., sex, age, smoking history, function of the primary organs, and biochemical assays) were introduced as variables in a new model and analyzed in the same way. Their performance can be demonstrated in PCA and OPLS-DA plots. Next, metabolic patterns were evaluated, and metabolic markers were screened and described based on semiquantitative data, ROC analysis, odds ratios (OR) (18), and their relations to well-recognized cardiovascular disease risk factors. Considering that plasma cells affect plasma metabolites most directly, the connection between plasma cells and metabolites is briefly explained in the discussion.

## Materials and Methods

### Human Plasma Collection

A total of 84 individual controls (social recruitment) and 146 chest pain cases highly suspected of MI were recruited from October 2017 to March 2018. (Exclusions include coma, fever, NYHA IV heart failure, hepatic decompensation, renal failure, cancers, and uncontrolled endocrine and hematological diseases.) After routine diagnostic procedures, including ECGs, infarction biomarkers, and coronary angiography (or coronary CT angiography, CCTA), 85 were later confirmed to be non-ST-elevation myocardial infarction (NSTEMI) or ST-elevation myocardial infarction (STEMI) as the MI cases (MIs), and the remaining 61 were confirmed to be non-MI chest pain cases (non-MIs). All the non-MIs include 34 unstable angina (UA) cases and 27 other non-MI cardiac cases (non-MICs), including myocarditis, valvular heart diseases, atrial fibrillation, etc (**Figure 3A**). The blood samples of the patients were collected not longer than 6 h since the onset of chest pain symptoms, before reperfusion therapy.

The study followed the principles outlined in the Declaration of Helsinki, and informed written consent was given prior to the inclusion of subjects in the study. The study was also under the guidance and supervision of the Ethics Committee of the First Affiliated Hospital of Nanjing Medical University (*Lot number: 2018-SR-028*). The venous blood samples were collected from fasting state volunteers in EDTA-Na anticoagulated tubes in the morning. Within 2 h, blood samples were centrifuged at 1,000 g for 5 min, and each of the supernatant plasma was transferred to another tube, frozen at −80°C in a refrigerator. Before using the plasma samples, they were thawed by incubation at 37°C bath for 15 min, vortexed, and centrifuged at 650 g for 5 min.

### Equipment for Blood Examinations

The instrument blood count, SYSMEX model xn-10, is made in Hyogo, Japan. Both NT-proBNP and serum infarction markers (cTns) are analyzed in Roche Cobas 6000 (produced in Mannheim, Germany). The blood biochemical instrument is AU5800 from Beckman Coulter of the United States, produced in Shizuoka, Japan.

### Chemicals and Reagents

Stable isotope internal standard 5-^13^C-glutamine was purchased from Cambridge Isotope Laboratories (Andover, MA, USA). Myristic-1,2-^13^C2 acid, methoxamine hydrochloride (purity 98%), and pyridine (≥99.8% GC) were purchased from Sigma-Aldrich (St. Louis, MO, USA). N-methyl-trimethylsilyl-trifluoroacetamide (MSTFA) and 1% trimethylchlorosilane (TMCS) were provided by Pierce Chemical (Rockford, IL, USA). Methanol, acetonitrile, and *n*-heptane were of HPLC grade and obtained from Merck (Darmstadt, Germany). Purified water was produced by a Milli-Q system (Millipore, Bedford, MA, USA). Ammonium acetate (purity 98.0%) and ammonia solution (25%, w/w) were purchased from Aladdin (Shanghai, China) and Nanjing Chemical Reagent (Nanjing, China), respectively.

### Method S1 GC/MS Analysis, Instrumental Setting, and Parameters

The plasma samples were pretreated, extracted, and derivatized in a similar way to that reported previously (19). Briefly, an aliquot of plasma (50 μl) was added to 200 μl methanol (containing internal standard [^13^C2]-myristic acid, 2.5 μg, 12.5 μg/ml) for GC/MS analysis and vigorously vortex-extracted for 5 min, and then placed in a fridge at 4°C for 1 h. After centrifuging at 20,000 g for 10 min in the SORVALL Biofuge Stratos centrifuge (Sollentum, Germany), a 200-μl aliquot of the supernatant was transferred into a GC vial and evaporated to dryness in a SpeedVac concentrator (Thermo Fisher Scientific, Savant™ SC250EXP, Holbrook, USA).

For GC/MS analysis, the dried plasma samples were then methoxymated, where 30 μl of 1% methoxyamine pyridine solution was added to the residue and incubated for 16 h at room temperature. Then, the analytes were trimethylsilylated using 30 μl of MSTFA containing 1% v/v trimethylchlorosilane (TMCS) as a catalyst. After trimethylsilylation for 1 h, 30 μl of *n*-heptane containing methyl myristate (30 μg/ml) was added into each GC vial as external standard to monitor the stability of GC/S instruments. The final mixture (90 μl in total) was vortexed for 1 min and was then ready for GC/MS analysis.

The derivatized samples were analyzed using gas chromatography coupled to a mass spectrometer (Shimadzu GCMS-QP2010 Ultra, Kyoto, Japan) equipped with an automatic sampler (Shimadzu AOC-20i, Kyoto, Japan). A 0.5-μl sample aliquot was injected into a liner connected with the Rtx-5MS capillary column (0.25 mm × 30 m × 0.25 μm, Restek, PA, USA) in split mode (split ratio 8:1). The injector temperature was set at 250°C. The septum purge was turned on with a flow rate of 6.0 ml/min. Helium was used as the carrier gas at a flow rate of 1.5 ml/min. The column temperature was initially maintained at 80°C for 5 min, then raised to 300°C at a rate of 20°C/min, and held for 5 min. The mass spectrometer ion source temperature was 220°C, and ionization was achieved with a 70-eV electron beam. Mass spectra were detected at −1,570 V, obtained from *m*/*z* 50 to 700 in a full scan mode, with each run of 19 min and a solvent cutting acquisition at 4.5 min. The quality control (QC) samples were prepared for the pool of plasma, with the same preparation procedure as mentioned above. To minimize systematic variations, all samples were analyzed at random order, with QC samples inserted.

### LC/MS Analysis, Instrumental Setting, and Parameters

The plasma samples were pretreated and extracted in the same way as in GC/MS analysis with a few modifications, which used the other internal standard of 5-^13^C-glutamine dissolved in methanol at 15 μg/ml. After vortexing and centrifugation, the final supernatant was transferred into an LC vial.

After evaporation, the residue was redissolved with 100 μl distilled water and centrifuged at 18,000 g for 5 min. Finally, 80 μl supernatant was transferred to an LC vial, and 10 μl was injected for UPLC-QTOF/MS analysis. Similarly, the QC samples were inserted and analyzed to check the stability of the system.

The HPLC-QTOF/MS analysis was carried out as previously reported (20). The chromatographic separation of the analyses was achieved with an Amide XBridge HPLC column (3.5 μm; 4.6 mm × 100 mm; Waters, USA). The column temperature was set to 40°C. The HPLC system consisting of a LC-30A binary pump, a SIL-30AC autosampler, and a CTO-30AC column oven (Shimadzu, Japan) was coupled with a hybrid quadrupole time-of-flight tandem mass spectrometer (AB SCIEX TripleTOF® 5600, Foster City, CA). The mobile phase was composed of 5 mM ammonium acetate in ultrapure water (pH = 9.0 ± 0.1 with ammonia) plus 5% acetonitrile (solvent A) and acetonitrile (solvent B). The mobile phase was delivered at 0.4 ml/min using a solvent gradient as follows: 0–3 min, 85% B; 3–6 min, 85–30% B; 6–15 min, 30–2% B; 15–18 min, 2% B; 18–19 min, 2–85% B; and 19–26 min, 85% B. A Turbo V electrospray ionization (ESI) was used in MS detection with negative ion modes. In the ESI source, parameters were set as follows: gas 1 pressure at 50 psi, gas 2 at 30 psi, and curtain gas at 30 psi; ion spray voltage was set at −4,500 V; turbo spray temperature was set at 500°C. Metabolic features were scanned in time-of-flight mass spectrometry over *m*/*z* 50–1,000, and the product ions were scanned over *m*/*z* 50–900, with declustering potential at −100 V and a collision energy at −35 V. The detected ions were all calibrated with the accurate masses of the reference standards containing amino-dPEG®4-acid (MW265.30, CAS: 663921-15-1), amino-dPEG®6-acid (MW353.41, CAS: 905954-28-1), amino-dPEG®8-acid (MW441.51, CAS: 756526-04-2), amino-dPEG®12-acid (MW617.72, CAS: 756526-07-4), and sulfinpyrazone (MW404.48, CAS: 57-96-5), for every eight samples.

### Multivariate Statistical Analysis

Missing data were excluded before the analysis. After normalization against the IS, the data were evaluated using SIMCA-P 14.1 software (Umetrics, Umeå, Sweden) (21). Principal component analysis (PCA), partial least square to latent structure discriminant analysis (PLS-DA), and orthogonal PLS-DA (OPLS-DA) models were built and plotted to show the clustering or separation of samples from different groups. For PLS-DA modeling, samples from the different groups were classified such that all samples were divided into different groups (e.g., MIs, non-MIs, controls, etc.) as the qualitative “dummy” variables, *Y*. The goodness of fit for the models was evaluated using three quantitative parameters: *R*^2^*X* and *R*^2^*Y* are the explained variation in *X* and *Y*, respectively, and *Q*^2^*Y* is the predicted variation in *Y*. Permutation test was assessed for model validation, where a higher level of *R*^2^*Y* and *Q*^2^*Y* and a lower value of the intercept of *R*^2^ (lower than 0.2) and *Q*^2^ (lower than 0.0) suggested good model and prediction ability.

### Discriminant Metabolites and Statistical Analysis

After normalization against the IS, all the semiquantitative data from both GC/MS and UPLC-QTOF/MS were logarized so that the state probabilities of the data queue tended to a normal distribution. The discriminant metabolites between groups were screened and chosen based on variable importance (VIP) using SIMCA-P 14.1 and the independent sample *t*-test of the logarized data using SPSS (version 23.0, SPSS Inc., Chicago, IL, USA). Fold change is calculated using raw but normalized data.

Metabolic pathway enrichment and topology analysis was performed online, using MetaboAnalyst 3.0 (https://www.metaboanalyst.ca/). The KEGG ID of discriminatory compounds was uploaded and embedded in human pathway library for pathway analysis and hypergeometric tests, with the pathway analysis algorithms of Fisher's exact test, topology algorithms of relative betweenness centrality, and KEGG pathway library version of *Homo sapiens*.

For data inconformity with normal distribution from clinical assaying, a non-parametric test (Mann–Whitney *U*-test, two-sided) was employed to evaluate statistical significance. ROC analysis and (adjusted) OR calculations were performed using SPSS as well. Before computing the OR value, the logarized data of a metabolite (*i*) for each subject of OR*i* were normalized by subtracting the mean value of ORmean within this group, and then divided by the standard deviation (SD) within the group, shown as the normalized ORs = (OR*i* – ORmean)/SD.

### Transcriptomics Database

We studied transcriptomics data from the GEO database. The human myocardial infarction plasma data are from GSE48060 (https://www.ncbi.nlm.nih.gov/geo/query/acc.cgi?acc=GSE48060) and GSE103182 (https://www.ncbi.nlm.nih.gov/geo/query/acc.cgi?acc=GSE103182). Mice myocardial data are from GSE775 (https://www.ncbi.nlm.nih.gov/geo/query/acc.cgi?acc=GSE775). The data matrix was directly extracted by GEOquery in R 4.0.3. Limma package was used to produce false discovery rate (FDR), fold change (FC), and *p*-value.

## Results

### Clinical Descriptions of Control, MI, and Non-MI Cases

Tables 1, 2 show the medical records and basic laboratory tests of the volunteers. Generally, higher glucose, AST, lactate dehydrogenase (LDH), hydroxybutyrate dehydrogenase (HBDH), and CK levels and lower albumin (ALB) and Ca^2+^ concentrations were detected in patients with chest pain. Among the 146 patients with cardiac chest pain, ~65% had taken aspirin and statin treatments before blood collection. As a result, total cholesterol (TC), triglycerides (TG), low-density lipoprotein cholesterol (LDL-C), and high-density lipoprotein cholesterol (HDL-C) levels were all lower in chest pain inpatients than the controls.

**Table 1:** Sample characteristics: controls vs. all the chest pain cases.

**Clinical concerns**	**Variables**	**Controls**	**Chest pain cases**	**Statistics**
		***n* = 84**	***n* = 146**	***p*-values**
Demographics	Male	56	99	0.26
	Age (years)	50.25 ± 1.76	59.28 ± 1.83	0.00
Cardiac risk factors	Hypertension	32	83	0.00
	Diabetes	8	36	0.00
	TC (mmol/L)	4.93 ± 0.15	4.08 ± 0.11	0.00
	TG (mmol/L)	1.43 ± 0.12	1.51 ± 0.08	0.17
	LDL-C (mmol/L)	3.15 ± 0.11	2.70 ± 0.08	0.00
	HDL-C (mmol/L)	**1.31 ± 0.04**	**0.95 ± 0.02**	0.00
	LPa (mg/L)	**349.35 ± 51.45**	**339.39 ± 27.47**	0.65
	Tobacco use	8	43	0.00
	Drinking history	0	16	0.00
Cardiovascular medications	Aspirin	1	88	0.00
	Statin therapy	6	94	0.00
	β-Blockers	0	7	0.00
Prior cardiovascular disease	2	22	0.01
Biochemical items	ALT (U/L)	27.15 ± 2.48	39.33 ± 2.90	0.00
	AST (U/L)	26.31 ± 1.45	**77.35 ± 9.78**	0.00
	ALP (U/L)	78.20 ± 2.19	86.16 ± 2.26	0.03
	GGT (U/L)	30.69 ± 2.75	45.18 ± 3.80	0.02
	LDH (U/L)	169.30 ± 3.85	**398.72 ± 32.22**	0.00
	CK (U/L)	119.30 ± 11.27	**480.97 ± 78.58**	0.94
	HBDH (U/L)	107.80 ± 3.87	**297.38 ± 28.85**	0.00
	TBIL (μmol/L)	14.36 ± 0.56	13.74 ± 0.71	0.03
	DBIL (μmol/L)	4.51 ± 0.28	4.96 ± 0.26	0.83
	IBIL (μmol/L)	9.16 ± 0.42	8.77 ± 0.48	0.05
	TP (g/L)	70.80 ± 0.47	61.12 ± 0.48	0.00
	ALB (g/L)	44.68 ± 0.54	**35.80 ± 0.36**	0.00
	GLB (g/L)	25.78 ± 0.48	25.33 ± 0.39	0.14
	ALB/GLB	1.79 ± 0.05	1.45 ± 0.03	0.00
	GLU (mmol/L)	5.77 ± 0.13	6.01 ± 0.22	0.23
	Urea (mmol/L)	5.13 ± 0.18	7.37 ± 0.52	0.00
	Cr (μmol/L)	71.14 ± 2.00	102.13 ± 11.16	0.00
	UA (μmol/L)	325.57 ± 9.61	378.91 ± 13.24	0.04
	Ca (mmol/L)	2.36 ± 0.02	**2.17 ± 0.01**	0.00

*Values are presented as mean ± SE. The Mann–Whitney U-test was applied to produce p-value. Bold values are abnormal findings of the test results*.

**Table 2:** Sample characteristics: MI vs. non-MI chest pain cases.

**Clinical concerns**	**Variables**	**Controls**	**Chest pain cases**		**Statistics**
		**(*n* = 84)**	**Non-MI (*n* = 61)**	**MI (*n* = 85)**	***p*-values**
Demographics	Male	56	32	67	0.00
	Age (years)	50.25 ± 1.76	60.18 ± 2.42	65.08 ± 1.60	0.12
Cardiac risk factors	Hypertension	32	32	51	0.28
	Diabetes	8	8	28	0.01
	Tobacco use	8	8	35	0.00
	TC (mmol/L)	4.93 ± 0.15	3.69 ± 0.12	4.28 ± 0.15	0.01
	TG (mmol/L)	1.43 ± 0.12	1.26 ± 0.09	1.65 ± 0.11	0.02
	LDL-C (mmol/L)	3.15 ± 0.11	2.40 ± 0.09	2.86 ± 0.11	0.01
	HDL-C (mmol/L)	1.31 ± 0.04	**0.96 ± 0.03**	**0.94 ± 0.03**	0.70
	LPa (mg/L)	**349.35 ± 51.45**	**334.55 ± 47.64**	**341.96 ± 33.82**	0.56
Prior cardiovascular disease	2	12	10	0.24	
Serum biomarkers	cTnT (ng/ml)	–	**756.13 ± 242.59**	**1,559.30 ± 276.64**	0.56
	CK-MB (U/L)	–	24.98 ± 6.08	**32.10 ± 5.89**	0.68
	Mb (ng/ml)	–	59.30 ± 23.79	66.99 ± 15.32	0.46
Biochemical items	ALT (U/L)	27.15 ± 2.48	32.17 ± 4.59	43.14 ± 3.65	0.04
	AST (U/L)	26.31 ± 1.45	29.12 ± 2.91	**102.99 ± 14.10**	0.00
	LDH (U/L)	169.30 ± 3.85	213.05 ± 10.22	**497.43 ± 45.36**	0.00
	CK (U/L)	119.30 ± 11.27	86.83 ± 12.99	**690.51 ± 113.49**	0.00
	HBDH (U/L)	107.80 ± 3.87	132.79 ± 6.71	**384.89 ± 40.81**	0.00
	TP (g/L)	70.80 ± 0.47	**61.95 ± 0.71**	**60.68 ± 0.62**	0.18
	GLU (mmol/L)	5.77 ± 0.13	5.49 ± 0.22	**6.28 ± 0.32**	0.06
	Ca (mmol/L)	2.36 ± 0.02	2.20 ± 0.02	**2.16 ± 0.02**	0.11

*Values are presented as mean ± SE. The Mann–Whitney U-test was applied to produce p-value between MI and non-MI chest pain cases. Bold values are abnormal findings of the test results*.

**Figure 1:**
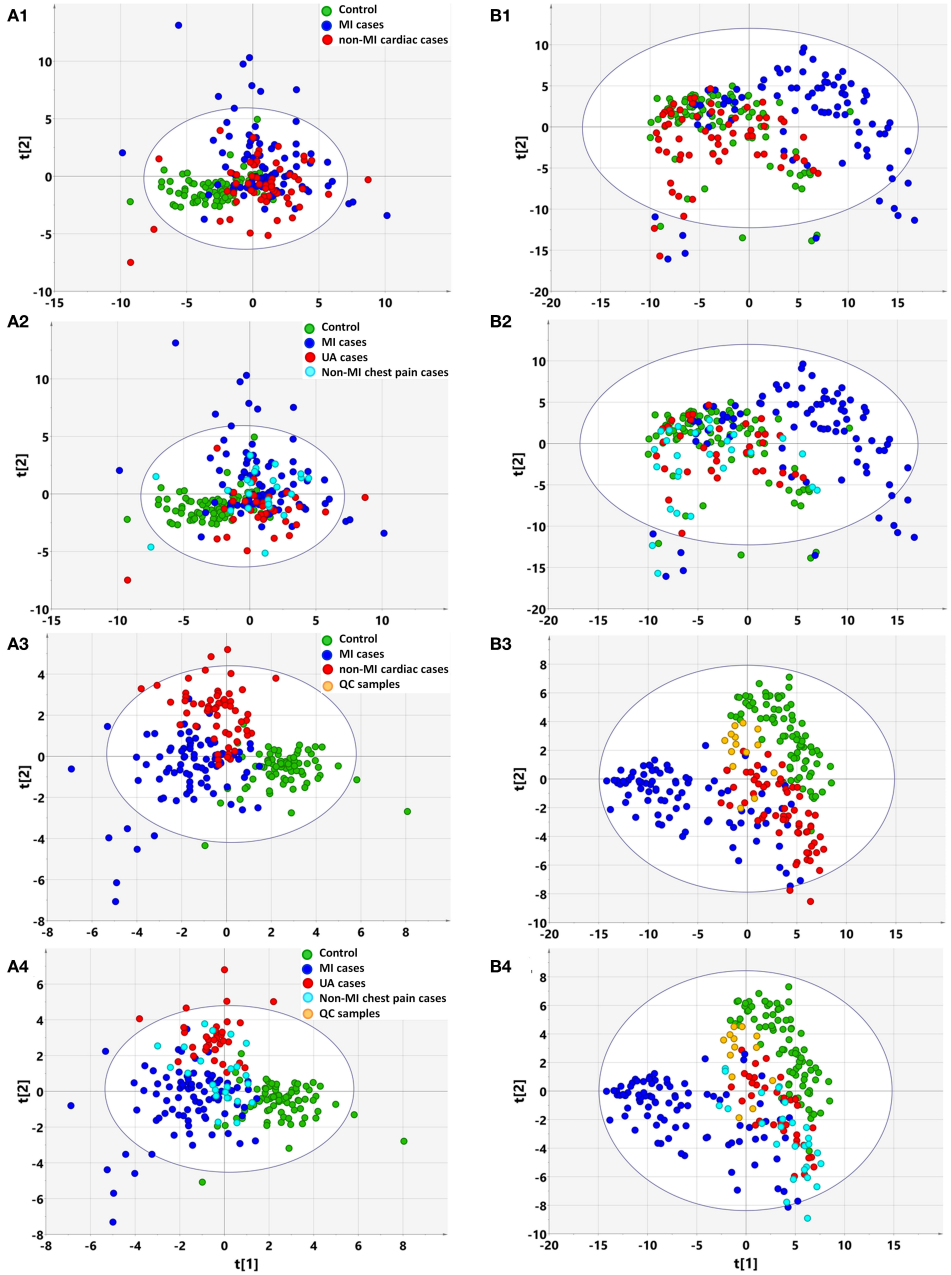
Multivariate statistical analysis differentiates the groups of myocardial infarction (MI) cases, non-MI chest pain cases, and controls based on clinical information **(A)** and metabolomic data **(B)**, respectively. (1) Non-supervised PCA modeling displays the original similarity of the three groups: MI cases (*n* = 85), non-MI chest pain cases (*n* = 61), and controls (*n* = 84), without arbitrary grouping. (2) Non-supervised PCA modeling displays the original similarity of the four groups: MI cases, unstable angina (UA), other non-MI cardiac cases, and controls, without arbitrary grouping. **(A1,A2)** PC1: *R*^2^*X*(cum) = 0.0895, *Q*^2^(cum) = 0.0106; PC2: *R*^2^*X*(cum) = 0.167, *Q*^2^(cum) = 0.0356. **(B1,B2)** PC1: *R*^2^*X*(cum) = 0.205, *Q*^2^(cum) = 0.189; PC2: *R*^2^*X*(cum) = 0.310, *Q*^2^(cum) = 0.274; PC3: *R*^2^*X*(cum) = 0.386, *Q*^2^(cum) = 0.338. (3) Supervised PLS-DA modeling with the three groups: MI cases, non-MI chest pain cases, and controls. **(A3)** PC1: *R*^2^*X*(cum) = 0.073, *R*^2^*Y*(cum) = 0.339, *Q*^2^(cum) = 0.270; PC2: *R*^2^*X*(cum) = 0.128, *R*^2^*Y*(cum) = 0.544, *Q*^2^(cum) = 0.362; PC3: *R*^2^*X*(cum) = 0.174, *R*^2^*Y*(cum) = 0.635, *Q*^2^(cum) = 0.394. Permutation tests with the intercepts of *R*^2^ < 0.23, *Q*^2^ < −0.20. **(B3)** PC1: *R*^2^*X*(cum) = 0.184, *R*^2^*Y*(cum) = 0.215, *Q*^2^(cum) = 0.207; PC2: *R*^2^*X*(cum) = 0.249, *R*^2^*Y*(cum) = 0.400, *Q*^2^(cum) = 0.377; PC3: *R*^2^*X*(cum) = 0.315, *R*^2^*Y*(cum) = 0.547, *Q*^2^(cum) = 0.512. Permutation tests with the intercepts of *R*^2^ < 0.10, *Q*^2^ < −0.05. (4) Supervised PLS-DA modeling with the four groups: MI cases (*n* = 85), UA (*n* = 34), other non-MI cardiac cases (*n* = 27), and controls (*n* = 84). **(A4)** PC1: *R*^2^*X*(cum) = 0.073, *R*^2^*Y*(cum) = 0.259, *Q*^2^(cum) = 0.198; PC2: *R*^2^*X*(cum) = 0.132, *R*^2^*Y*(cum) = 0.397, *Q*^2^(cum) = 0.269; PC3: *R*^2^*X*(cum) = 0.173, *R*^2^*Y*(cum) = 0.492, *Q*^2^(cum) = 0.289. Permutation test with the intercepts of *R*^2^ < 0.180, *Q*^2^ < −0.15. **(B4)** PC1: *R*^2^*X*(cum) = 0.183, *R*^2^*Y*(cum) = 0.169, *Q*^2^(cum) = 0.163; PC2: *R*^2^*X*(cum) = 0.254, *R*^2^*Y*(cum) = 0.300, *Q*^2^(cum) = 0.277; PC3: *R*^2^*X*(cum) = 0.317, *R*^2^*Y*(cum) = 0.422, *Q*^2^(cum) = 0.387. Permutation test with the intercept of *R*^2^ < 0.11, *Q*^2^ < −0.05.

Based on clinical parameters (listed in Table 1 “variables”), including “biochemical items,” “demographics,” and “cardiac risk factors,” an unsupervised PCA score plot was created. The model indicated a few outliers when the samples were either divided into three (controls, MIs, non-MIs) or four groups (controls, UA, MIs, and other non-MICs), and each of the groups generally overlapped with the others (Figures 1A1,A2). However, a supervised PLS-DA revealed a visible separation of the groups with only a little overlap when the samples were divided into three groups, i.e., MIs, non-MIs, and controls. When the samples were divided into four groups (MIs, UAs, other non-MICs, and controls), the controls, UAs, and MIs were fairly well-separated, but the other non-MICs primarily showed overlaps with UAs and MIs (Figures 1A3,A4). These findings suggest that the model was not powerful at differentiating non-MICs from MIs and UAs based on basic laboratory tests and history examinations.

### Plasma Metabolomic Description of Chest Pain Individuals by PCA and OPLS-DA Plots

GC/MS and LC/MS analysis of the plasma samples aligned the metabolites in typical chromatograms (Supplementary Figures 1, 2). Deconvolution of the GC/MS chromatograms produced 135 independent peaks from the plasma samples, 83 of which were authentically identified as metabolites; LC/MS produced 279 peaks, and 76 metabolites were identified (Supplementary Tables 1, 2). Quantitative data were acquired for each metabolite in the plasma samples of the control, MI, UA, and other non-MI cardiac cases.

Based on the metabolomic data derived from GC/MS and LC/MS analysis, the PCA score plot again showed a few outliers when the samples were divided into three or four groups, as indicated above (**Figure 3A**). Unlike with the clinical data, unsupervised PCA analysis of metabolomic data showed that the majority of MIs deviated from the others, regardless of whether the three or four groups were defined, although the control, non-MICs, and UAs overlapped with each other to some extent (Figures 1B1,B2). This suggests that the identified plasma substances can naturally detect the difference between MI and other samples (including healthy controls, UA) and there are MI marker metabolites in the metabolite profile.

The supervised PLS-DA model revealed that samples from each group clustered closely and anchored away from the other groups when the samples were divided into three groups (Figure 1B3). When the samples were divided into four groups, the majority of MIs and controls clustered separately, while the UAs and non-MICs primarily overlapped with each other, with a minority overlapping with MIs and controls (Figure 1B4). The distant separation of MIs from the other groups suggested distinctly different metabolic patterns between MIs and the groups of UA and non-MICs, while the overlapping of the groups suggested similar plasma metabolic patterns between UA and the other non-MICs. In general, metabolomic data better characterized MIs than history examinations and laboratory tests, and the score plot of non-MI chest pain cases (including UA and non-MI cardiac cases) indicated that they had moderate metabolic perturbation relative to the MI cases because they anchored between MI and the controls (Figure 1B3). The above data suggest that subgroups of MI can be recognized by multivariate analysis of identified plasma metabolites more effectively than by routine clinical parameters.

### Pathway Analysis of Differential Metabolites

**Figure 2:**
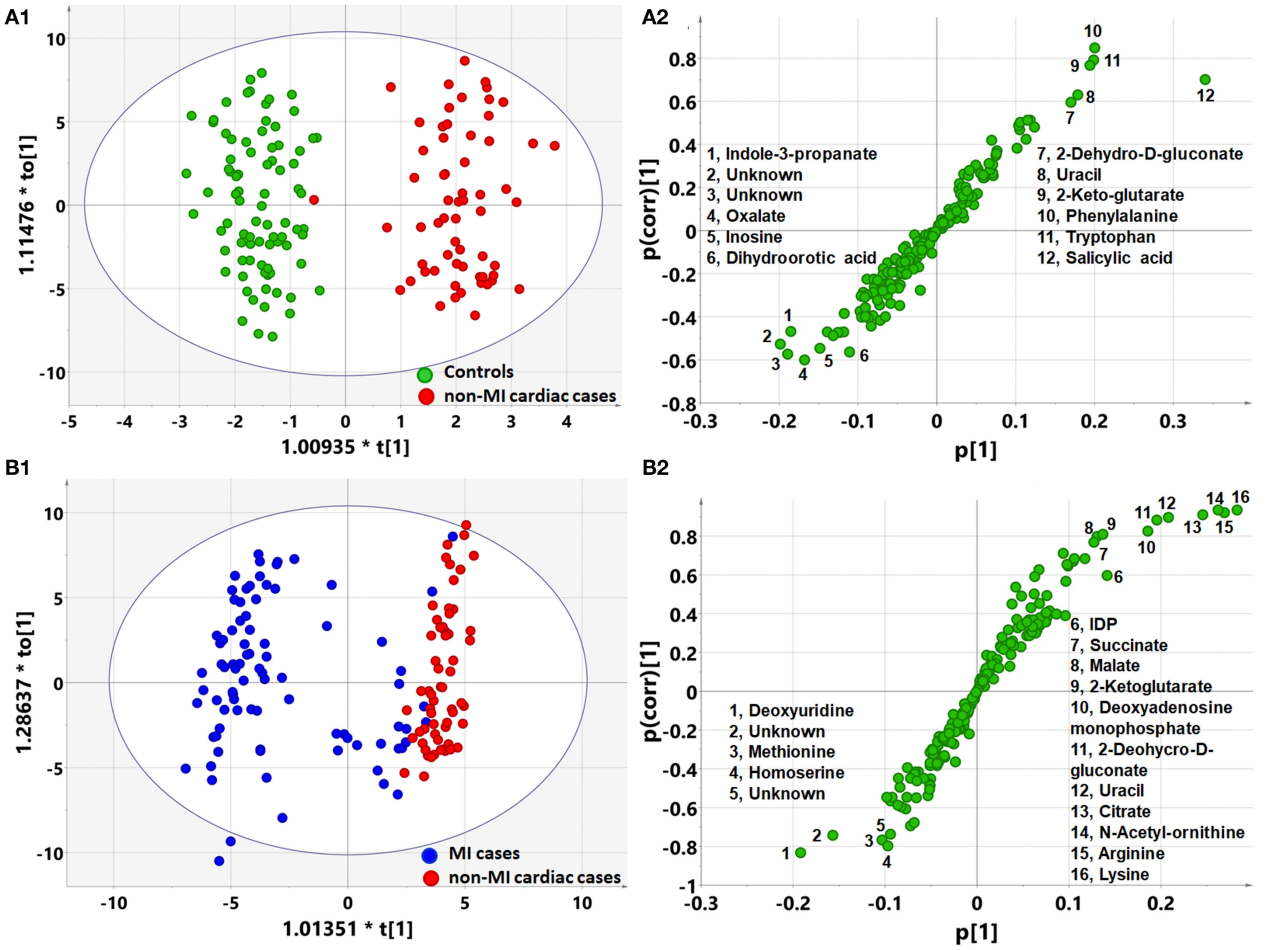
Orthogonal partial least square to latent structure discriminant analysis (OPLS-DA) modeling and S-plots delineate different metabolic phenotypes and potential markers of MI and non-MI cardiac cases. **(A1)** OPLS-DA model differentiating non-MI cardiac cases (*n* = 61) from the controls (*n* = 84). **(A2)** S-plot highlights the potential markers of the non-MI cardiac cases. **(B1)** OPLS-DA model differentiating MI cases (*n* = 85) from non-MI cardiac cases (*n* = 61). **(B2)** S-plot highlights the potential markers of the MI cases.

OPLS-DA analysis showed a different metabolomic pattern of the non-MIs from the controls (Figure 2A1). Statistical analysis suggested 50 discriminant metabolites (*p* < 0.05) that differentiated non-MI chest pain inpatients from the controls (Table 3, S-plot: Figure 2A2). Similarly, MI cases primarily showed different metabolomic patterns from non-MIs (Figure 2B1). According to the statistical analysis and the VIP values, 54 discriminant metabolites were identified between MIs and non-MIs (Table 3, S-plot: Figure 2B2).

**Table 3:** List of discriminant metabolites: non-MIs vs. controls and MIs vs. non-MIs.

**Differential metabolites**	**Controls (*****n*** **= 84)**		**MI cases (*****n*** **= 85)**		**Non-MI cases (*n* = 61)**	**MI vs. non-MI**		**Non-MI vs. Con**		
	**Mean**	**SE**	**Mean**	**SE**	**Mean**	**SE**	**FC**	***t*-test**	**FC**	***t*-test**
Deoxyuridine	26,212	9,533	1,025,278	150,576	16,840	555	60.885	^***^	0.642	/
Adenosine phosphosulfate	51,723	4,141	99,104	8,542	42,213	3,793	2.348	^***^	0.816	/
Deoxyadenosine monophosphate	310,296	7,783	102,462	13,401	311,056	7,624	0.329	^***^	1.002	/
Guanosine diphosphate	7,379	469	27,614	4,153	6,648	530	4.154	^***^	0.901	/
Inosine 2′-phosphate	14,875	683	37,785	4,687	14,988	809	2.521	^***^	1.008	/
Adenosine monophosphate	20,124	1,044	25,648	1,689	19,902	1,016	1.289	^***^	0.989	/
Hypoxanthine	705,034	24,192	373,454	29,693	734,901	52,065	0.508	^***^	1.042	/
Glycolate	8,319	247	9,121	331	7,620	306	1.197	^**^	0.916	/
Methionine	113,239	2,830	335,994	27,792	109,192	2,631	3.077	^***^	0.964	/
Arginine	810,028	22,688	169,566	34,842	731,551	31,282	0.232	^***^	0.903	/
Valine	2,334,562	53,682	3,710,196	209,295	2,425,637	63,465	1.530	^***^	1.039	/
Citrulline	422,244	11,079	296,302	13,267	469,763	20,870	0.631	^***^	1.113	/
Shikimate	14,871	796	8,837	491	15,072	782	0.586	^***^	1.014	/
Ornithine	747,344	19,685	522,218	23,919	805,398	34,957	0.648	^***^	1.078	/
Alanine	402,892	10,006	663,687	37,026	446,350	16,079	1.487	^***^	1.108	/
Glycine	145,111	2,764	256,201	15,108	150,041	3,152	1.708	^***^	1.034	/
Homocysteine	7,517	382	20,169	2,293	7,693	504	2.622	^***^	1.023	/
Aspartate	155,030	10,889	194,713	12,674	123,523	10,287	1.576	^***^	0.797	/
Ribose	223,498	12,602	340,299	15,678	254,617	23,072	1.337	^**^	1.139	/
1-Monopalmitin	30,533	1,705	33,208	1,616	25,699	1,904	1.292	^**^	0.842	/
Glycerate	125,449	4,077	54,424	3,823	127,894	10,153	0.426	^***^	1.019	/
Citrate	6,687,162	203,004	1,395,841	280,056	6,186,805	304,233	0.226	^***^	0.925	/
NAD+	11,766	301	25,536	3,262	11,479	330	2.225	^***^	0.976	/
NADPH	5,344	269	35,996	7,862	4,157	192	8.659	^***^	0.778	###
Uracil	146,594	7,319	60,619	9,826	319,956	18,480	0.189	^***^	2.183	###
Xanthine	161,018	5,234	99,621	11,423	234,923	11,566	0.424	^***^	1.459	###
Adenosine	33,452	2,133	31,326	3,112	18,676	1,948	1.677	^***^	0.558	###
IDP	3,112,579	73,422	1,512,965	153,666	3,597,655	92,953	0.421	^***^	1.156	###
Adenine	15,026	2,519	13,092	2,292	5,543	393	2.362	^**^	0.369	###
Succinate	88,550	1,816	36,686	3,233	79,439	2,388	0.462	^***^	0.897	##
Malate	61,898	2,263	35,918	4,299	78,778	4,163	0.456	^***^	1.273	###
2-Ketoglutarate	33,628	1,314	21,806	2,264	78,634	4,864	0.277	^***^	2.338	###
Acetoacetate	132,245	2,671	235,115	13,087	160,911	7,415	1.461	^***^	1.217	###
Carbamoylphosphate	29,690	1,819	23,063	2,179	36,316	1,532	0.635	^***^	1.223	##
Dihydroorotate	33,819	1,003	31,876	2,619	22,310	579	1.429	^***^	0.660	###
Pantothenate	54,720	3,022	38,266	4,405	83,814	4,827	0.457	^***^	1.532	###
Phenylpyruvate	51,177	955	63,363	2,279	56,537	1,514	1.121	*	1.105	##
Cysteine	106,041	3,333	68,660	3,639	150,632	7,856	0.456	^***^	1.421	###
Isoleucine	1,694,252	47,827	4,794,207	400,231	2,264,556	105,757	2.117	^***^	1.337	###
Serine	187,997	4,449	345,414	14,192	211,966	5,374	1.630	^***^	1.127	###
Proline	805,696	27,760	1,281,948	56,175	912,165	3,390	1.405	^***^	1.132	#
Threonine	661,526	23,707	1,415,808	70,560	574,241	19,440	2.466	^***^	0.868	##
Phenylalanine	1,051,097	31,497	1,757,945	133,629	2,349,797	58,409	0.748	^***^	2.236	###
Glutamine	6,299,813	30,803	6,265,669	37,518	6,115,270	38,706	1.025	^**^	0.971	###
Histidine	3,756,407	54,216	2,876,183	73,183	3,370,631	66,123	0.853	^***^	0.897	###
Taurine	851,621	35,791	1,382,464	89,177	727,822	36,862	1.899	^***^	0.855	#
Lysine	1,733,874	38,073	505,537	102,029	2,002,743	55,294	0.252	^***^	1.155	###
N-acetylornithine	329,453	18,159	68,946	14,885	265,119	11,352	0.260	^***^	0.805	##
Cytosine	7,058	102	5,624	153	6,609	135	0.851	^***^	0.936	##
3-Phospho-serine	11,045	654	17,870	1,469	8,893	284	2.009	^***^	0.805	##
Homoserine	661,526	23,707	1,415,808	70,560	574,241	19,440	2.466	^***^	0.868	##
1-Monostearin	17,548	911	19,128	896	13,626	841	1.404	^***^	0.777	##
2-Dehydro-D-gluconate	47,777	2,930	21,494	3,999	90,849	4,764	0.237	^***^	1.902	###
Oxalate	44,511	2,459	48,639	3,025	27,020	2,378	1.800	^***^	0.607	###
Tryptophan	647,430	23,608	1,366,119	100,129	1,584,205	58,633	0.862	/	2.447	###
Glutamate	253,094	9,911	384,958	35,729	384,612	16,588	1.001	/	1.520	###
Hydroxyproline	26,782	1,974	17,148	1,951	17,583	2,030	0.975	/	0.657	##
Salicylic acid	4,870	1,788	72,167	12,248	57,320	7,503	1.259	/	11.771	###
Pyruvate	121,954	5,245	183,553	22,838	184,240	7,253	0.996	/	1.511	###
Homocysterate	11,932	344	14,822	1,109	13,267	310	1.117	/	1.112	##
Cystathionine	10,857	625	13,618	1,322	17,869	1,568	0.762	/	1.646	###
2-Hydroxybutyrate	279,219	12,481	421,962	23,064	405,608	28,986	1.040	/	1.453	###
3-Hydroxybutyrate	52,649	3,298	79,377	10,282	85,759	13,390	0.926	/	1.629	#
Gluconic acid	105,169	4,261	289,949	39,667	203,681	34,215	1.424	/	1.937	##
Indole-3-propanate	55,659	16,898	8,531	1,748	5,464	801	1.561	/	0.098	##
Glycerol	400,975	13,803	290,401	11,659	303,966	15,733	0.955	/	0.758	###
Cholesterol	822,261	17,789	690,461	14,851	684,060	16,272	1.009	/	0.832	###
Alpha-tocopherol	116,918	2,949	93,627	2,218	94,284	2,954	0.993	/	0.806	###
Deoxyadenosine	23,123	1,541	17,568	2,255	13,041	1,288	1.347	/	0.564	###
Thymine	544,496	17,903	518,803	20,345	457,002	25,215	1.135	/	0.839	##
Inosine	65,657	2,868	28,268	3,939	35,665	2,829	0.793	/	0.543	###
NADP+	24,365	1,068	23,264	1,803	19,968	712	1.165	/	0.820	###

The data were not logarized and expressed as mean ± SE. Fold change (FC) was calculated by the original, non-logarized data directly. Statistical significance was evaluated by using two-tailed t-test with equal variance, after ANOVA assessment of the variance. ^***^, ^**^, ^*^: p < 0.001, 0.01, 0.05, respectively, between MI and non-MI chest pain cases; ###, ##, #: p < 0.001, 0.01, 0.05, respectively, between non-MI chest pain cases and controls. “/” represents the statistical significance of p-values more than 0.05.

**Figure 3:**
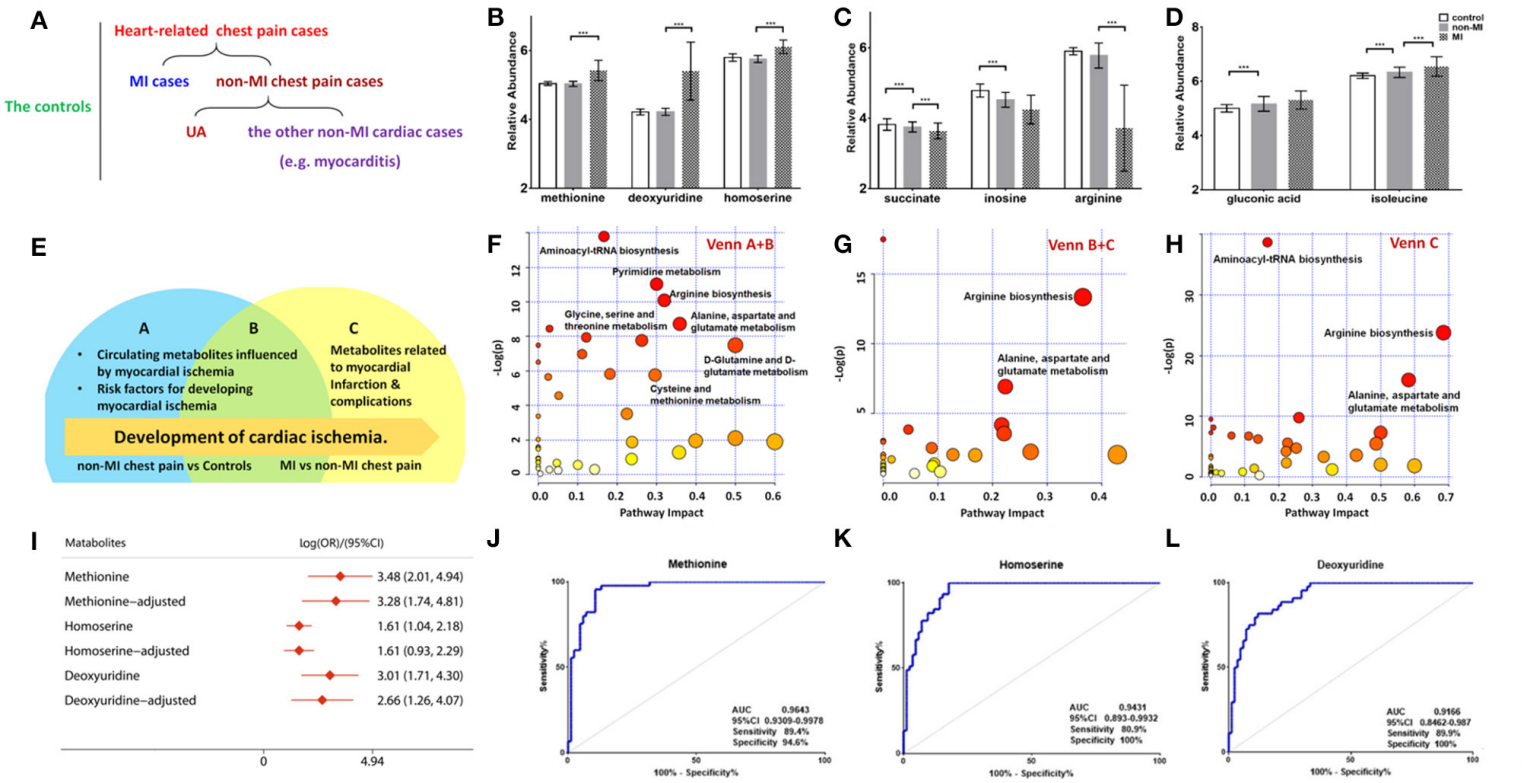
Differential metabolites and pathways involved in the MI group. **(A)** Samples are divided into three groups: control (*n* = 84), MI (*n* = 85) cases, and non-MI chest pain cases (*n* = 61); or four groups: control (*n* = 84), MI cases (*n* = 85), UA (*n* = 34), and the other non-MI cardiac cases (*n* = 27) when OPLS-DA analysis is applied. **(B)** Plasma methionine, deoxyuridine, and homoserine levels spike in the MI group. **(C)** Succinate, inosine, and arginine levels drop as CAD progresses to MI. **(D)** Gluconic acid and isoleucine levels increase as CAD progresses to MI (relative abundance is shown in logarized form: mean with SD, ***p < 0.05). **(E)** Venn diagram shows discriminant metabolites can be classified into regions A, B, and C. **(F)** Pathway analysis of differential metabolites in Venn A and B regions (control vs. non-MI chest pain cases). **(G)** Pathway analysis of differential metabolites in Venn C and B (MI vs. non-MI cases). There is a remarkable change of arginine biosynthesis and alanine, aspartate, and glutamate metabolism. **(H)** Pathway analysis of differential metabolites in Venn C. There is a remarkable change of arginine biosynthesis and alanine, aspartate, and glutamate metabolism. **(I)** Unadjusted and adjusted (adjusted for age, gender, LDL-C, HDL-C, smoking/diabetic/hypertensive history) odds ratio of methionine, deoxyuridine, and homoserine. **(J)** Receiver operating characteristic curve (ROC) analysis of methionine [area under the ROC curve (AUC) 96.43%, sensitivity 89.4%, specificity 94.59%]. **(K)** ROC analysis of homoserine (AUC 94.31%, sensitivity 80.9%, specificity 100%). **(L)** ROC analysis of deoxyuridine (AUC 91.66%, sensitivity 80.9%, specificity 100%) (***p < 0.001).

Of the 50 metabolites differentiating non-MIs from the controls, the levels of gluconic acid and isoleucine were higher in non-MIs, while succinate, inosine, and arginine were lower, and all the above metabolites deviated further in MIs (Figures 3C,D). These findings indicate that the above metabolites are involved in the development of cardiac damage (from averagely very small damage to severe infarction).

Although glycerol, salicylic acid, and deoxyadenosine showed significant differences between the non-MI and control groups, they had no significant difference between the MI and non-MI cardiac groups. These are, thus, suggested as markers of non-MI chest pain. In addition to endogenous metabolites, we found that salicylic acid, an exogenous metabolite, also characterized the group of chest pain cases. Salicylic acid is the primary metabolite of aspirin, and a review of inpatient information and clinical data revealed that a large portion of chest pain patients had taken aspirin for the management of CAD.

Moreover, deviated levels of dU, methionine, homoserine, etc. were only observed in MI cases (vs. non-MI chest pains), and no significant difference was observed between the controls and non-MIs, indicating their association with the development of MI (Figure 3B).

The differential metabolites between acute coronary syndrome (ACS, including UA and MI) and healthy individuals (ACS vs. control) can play roles in lipid plaque rupture; thus, they have the potential in alerting the occurrence of ACS. We found 127 identified differential metabolites. Supplementary Table 3 lists the top 20 metabolites (sort by *p*), and most of them are amino acids. KEGG analysis highlighted ACS's upregulated cysteine and methionine metabolism, phenylalanine metabolism, and synthesis and degradation of ketone bodies pathway and downregulated arginine biosynthesis, purine metabolism, and pyrimidine metabolism (FDR < 0.05). The metabolites with large FCs are salicylic acid, dU, guanosine diphosphate, gluconic acid, homocysteine, NADPH, methionine, tryptophan, mannopyranose, and isoleucine (top 10, FC > 1, *p* < 0.05). The metabolites with the smallest FCs (*p* < 0.05, FC < 1) are succinate, isopentenyl diphosphate (IDP), glutamine, inosine, uracil, citrate, lysine, N-acetylornithine, indole-3-propanate, and alanine.

A Venn diagram was created to show the discriminant metabolites between MI and non-MIs and those between non-MIs and controls. The overlapping region (B) in the Venn diagram (Figure 3E) lists the metabolites screened out in both of the two comparison groups, suggesting that they were most likely the risk factors or markers of the occurrence and development of MI, reflecting homeostatic disturbance induced by myocardia hypoxia. Figure 3F shows the pathway analysis of the metabolites in the Venn A+B region (control vs. non-MI), and Figure 3G shows the pathway analysis of discriminant metabolites in the Venn B+C region (MI vs. non-MI). Generally, arginine biosynthesis and pyrimidine metabolism were the most significantly altered metabolic pathways in non-MI chest pain patients' plasma compared with that in healthy individuals. Enrichment and pathway analysis for the metabolites of the Venn C area by MetaboAnalyst showed that arginine biosynthesis (*p* < 0.01, FDR < 1%) was the most altered metabolic pathway (Figure 3H). Alanine, aspartate, and glutamate metabolism (*p* < 0.01, FDR < 1%) deserve attention in MI as well.

**Figure 4:**
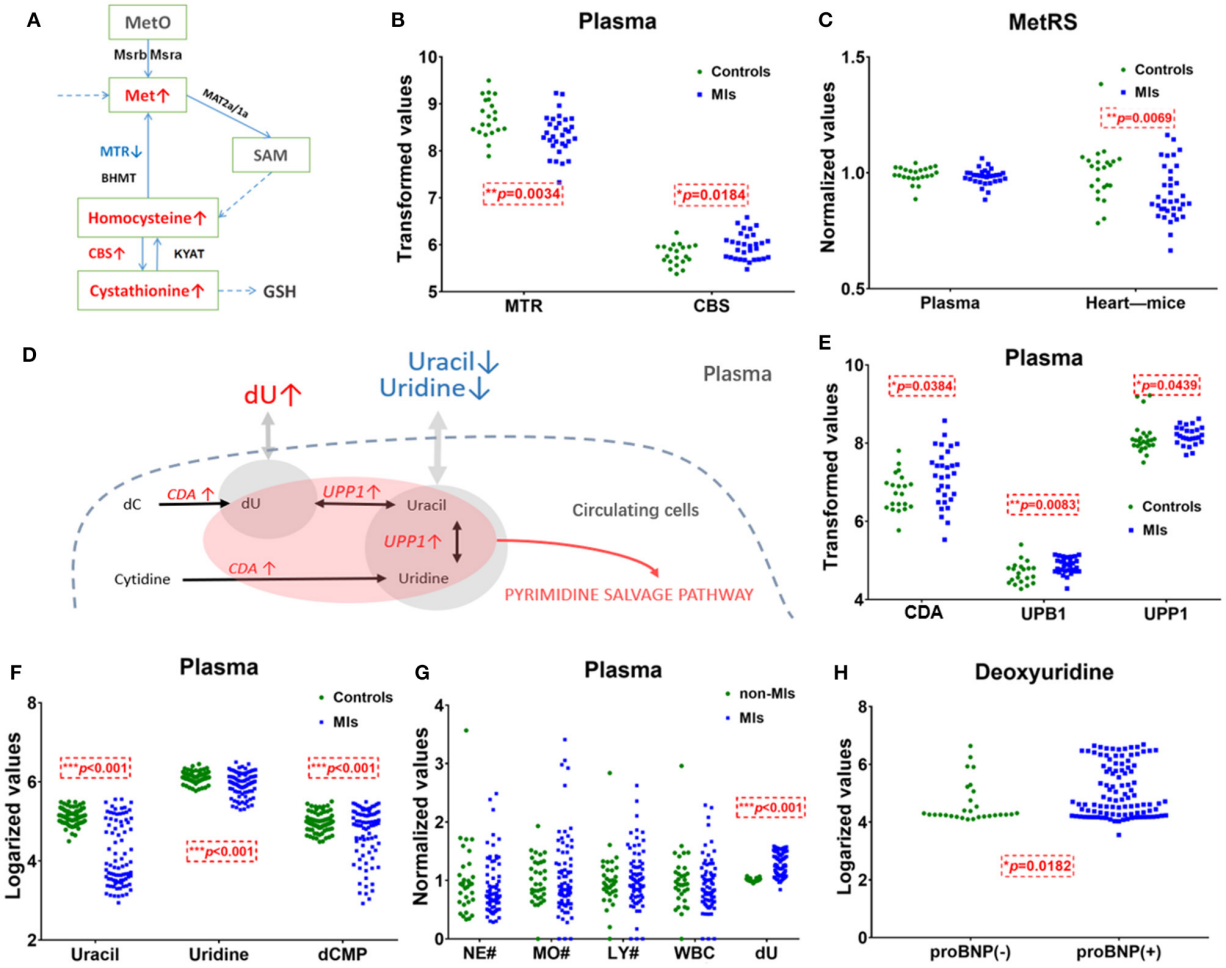
Methionine and deoxyuridine (dU)-related pathways in plasma. **(A)** Methionine (Met) abundance-related pathway in plasma. **(B)** The Met-related enzyme *MTR* is significantly* decreased and *CBS* is significantly higher in MI cases (*n* = 31) compared with healthy individuals (*n* = 21). **(C)** The Met-related enzyme *MetRS*(*MARS2*) abundance in the myocardium of murine MI models (*n* = 36) is downregulated* compared with control (*n* = 23), while human plasma *MetRS* remains unchanged. **(D)** dU-centered metabolism in plasma cells. **(E)** dU-related enzymes *CDA, UPB1*, and *UPP1* are significantly* higher in the MI group (*n* = 31) plasma compared with those in healthy individuals (*n* = 21). **(F)** dU-related intermediates uracil, uridine, and dCMP significantly* dropped in MI cases (*n* = 85) compared with those in healthy individuals (*n* = 84). **(G)** Neutrophils, monocytes, lymphocytes, and white cell counts of non-MI cases (*n* = 34) and MI cases (*n* = 74). **(H)** dU is significantly* higher in the NT-proBNP(+) group (*n* = 104) than in the NT-proBNP(–) group (*n* = 29). **(A,D)**: *red* indicates upregulated metabolites or enzymes, *blue* indicates the downregulated, *black* indicates the unchanged, and *gray* indicates undetected metabolites. *Student's *t*-test, *p* < 0.05.

The pathway of pyrimidine metabolism was deranged in the MI cases, as shown by the dramatic changes in dU and uracil. Figure 4D shows metabolites and metabolic enzymes in the dU-related pathway.

### Methionine, dU (Deoxyuridine), and Homoserine Are the Main Markers for MI Occurrence

A combined biosignature of homoserine, IDP, and 2-ketoglutarate discriminated MI from non-MI chest pain inpatients with high accuracy [Supplementary Figure 3I, area under the ROC curve (AUC) = 0.98, sensitivity = 94.1%, specificity = 100%].

The potential capacity of each discriminant metabolite to diagnose MI (MI vs. non-MI chest pain cases) was assessed by ROC analysis. Notably, although pathway analysis did not draw our attention to the methionine-related metabolic module, methionine and homoserine showed their potential in distinguishing MI from non-MI cardiac cases. Homoserine (AUC = 0.94, specificity = 100%, sensitivity = 81%) was more specific for MI diagnosis but less sensitive than methionine (AUC = 0.96, specificity = 94.6%, sensitivity = 89.4%) (Figures 3J,K). dU also scored highly, with an AUC over 90% (Figure 3L). dU level is also significantly higher in the proBNP-positive group than in the proBNP-negative chest pain group (Figure 4H). Some other metabolites that showed MI diagnostic potential were 2-ketoglutarate, arginine, 2-dehydro-D-gluconate, uracil, etc (Table 4).

**Table 4:** Differential metabolites and the diagnostic potential between MI and non-MI chest pain cases.

**Differential metabolites**	**AUROC**	**95% CI**	**Sensitivity (%)**	**Specificity (%)**	**LogOR**	**95% CI**
Homoserine, IDP, and α-ketoglutarate	0.9810	0.9614–1.0000	94.10	100	3.02	2.12 to 3.93
Methionine	0.9643	0.9309–0.9978	89.40	94.60	3.48	2.01 to 4.94
Homoserine	0.9431	0.893–0.9932	80.90	100	1.61	1.04 to 2.18
α-Ketoglutarate	0.9390	0.8876–0.9905	100	0.00	−0.11	−0.28 to −0.03
Uracil	0.9166	0.8585–0.9747	100	0.00	−2.38	−3.45 to −1.32
Deoxyuridine	0.9166	0.8462–0.987	80.90	100	3.01	1.71 to 4.30
2-Dehydro-D-gluconate	0.9040	0.8362–0.9717	2.10	100	−1.69	−2.53 to −0.84
Cysteine	0.8976	0.8302–0.9651	100	0.00	−0.41	−0.61 to −0.20
Deoxyadenosine monophosphate	0.8976	0.8335–0.9618	100	0.00	−5.57	−8.61 to −2.53
IDP	0.8838	0.8124–0.9553	100	0.00	−5.15	−7.86 to −2.44
Glyceric acid	0.8758	0.8022–0.9493	100	0.00	−1.53	−2.08 to −0.98
Citrate	0.8568	0.7774–0.9362	100	0.00	−1.69	−2.50 to −0.88
Succinate	0.8562	0.7768–0.9357	100	0.00	−1.48	−2.14 to −0.83
Pantothenate	0.8332	0.7452–0.9213	100	0.00	−0.98	−1.36 to −0.59
Xanthine	0.8240	0.7312–0.9168	2.10	100	−1.06	−1.46 to −0.65
Arginine	0.8235	0.7319–0.9151	2.10	100	−0.82	−1.10 to −0.53

*Logarization transformation of the data was applied to the data before the calculation*.

cTnT, CK-MB, AST, LDH, and HBDH are well-recognized indicators involved in myocardial damage and infarction. As candidate markers of MI, methionine, dU, and homoserine were significantly and positively correlated with LDH, HBDH, and AST (Supplementary Figure 3B), but not with cTnT or CK-MB. Six metabolites, 2-hydroxybutyrate, 3-hydroxybutyrate, homocysteine, palmitic acid, stearic acid, and 1-monooleoylglycerol, were positively and significantly correlated with both cTnT and CK-MB (Supplementary Figure 3C).

Some other metabolites that showed good diagnostic potential were cysteine, 2-ketoglutarate, IDP, and uracil (Table 4).

### Traditional CAD Risk Factors and Cardiac Function Influence the Metabolic Pattern

Correlation analysis showed that methionine, homoserine, homocysteine, and dU were all affected by smoking history, but none was obviously perturbed by hypertension (Supplementary Table 4). Moreover, to assess the role of these metabolites as risk factors for the prediction of MI occurrence, OR values were calculated between the MI and non-MI groups. Homoserine, dU, and methionine had high scores (Table 4). After adjusting for age, sex, LDL-C, HDL-C, smoking/diabetes/hypertension history, and logOR^(MI/non−MI)^ of methionine, homoserine, and dU, all had ORs > 1 (MIs vs. non-MIs) (Figure 3I). In the subgroup analyses of smoking/non-smoking, hypertensive/normotensive, diabetic/non-diabetic, aged 45–54/55–65, and male/female, the means of homoserine, methionine, and dU were all higher in the MI cases (Table 5). It shows that higher methionine, homoserine, or dU plasma level increases the risk of chest pains being diagnosed as MI.

**Table 5:** Mean of deoxyuridine, methionine, and homoserine in certain subgroups of the controls, non-MIs, and MI cases.

**Risk factors**	**Subgroups**	**Deoxyuridine**	**Methionine**	**Homoserine**	**Non-risk factors**	**Subgroups**	**Deoxyuridine**	**Methionine**	**Homoserine**
Smokers	Controls	4.22	5.04	5.82	Non-smokers	Controls	4.24	5.05	5.8
	Non-MI cases	4.22	5.03	5.76		Non-MI cases	4.22	5.03	5.75
	MI cases	5.48^*^^,^^#^	5.44^*^^,^^#^	6.13^*^^,^^#^		MI cases	5.45^*^^,^^#^	5.43^*^^,^^#^	6.11^*^^,^^#^
Diabetic	Controls	4.22	5.04	5.8	Nondiabetic or insulin resistance	Controls	4.24	5.05	5.8
	Non-MI cases	4.2	5.03	5.75		Non-MI cases	4.21	5.04	5.73
	MI cases	5.44^*^^,^^#^	5.43^*^^,^^#^	6.12^*^^,^^#^		MI cases	5.34^*^^,^^#^	5.40^*^^,^^#^	6.09^*^^,^^#^
Hypertensive	Controls	4.24	5.05	5.8	Normotensive	Controls	4.22	5.04	5.8
	Non-MI cases	4.22	5.03	5.75		Non-MI cases	4.22	5.04	5.73
	MI cases	5.40^*^^,^^#^	5.42^*^^,^^#^	6.11^*^^,^^#^		MI cases	5.37^*^^,^^#^	5.42^*^^,^^#^	6.10^*^^,^^#^
Age 45–54	Controls	4.29	5.07	5.83	Age 55–65	Controls	4.23	5.03	5.83
	Non-MI cases	4.21	5.05	5.72		Non-MI cases	4.18	5.04	5.75
	MI cases	5.30^*^^,^^#^	5.37^*^^,^^#^	6.13^*^^,^^#^		MI cases	5.60^*^^,^^#^	5.45^*^^,^^#^	6.11^*^^,^^#^
Male	Controls	4.22	5.05	5.8	Female	Controls	4.28	5.05	5.82
	Non-MI cases	4.22	5.05	5.77		Non-MI cases	4.23	5	5.72
	MI cases	5.43^*^^,^^#^	5.44^*^^,^^#^	6.11^*^		MI cases	5.32^*^^,^^#^	5.35^*^^,^^#^	6.10^*^^,^^#^

* *MI vs. controls, p < 0.05;*

# *MI vs. non-MI, p < 0.05*.

*All data were logarized before calculating, and the result showed the means of each subgroup*.

As a clinical indicator of cardiac function in MI, positive NT-proBNP represents cardiac dysfunction. Methionine, homoserine, and deoxyuridine were further elevated in NT-proBNP-positive cases. Pathway analysis of the discriminant metabolites (Supplementary Table 5) between the NT-proBNP-positive and NT-proBNP-negative groups suggested that only arginine biosynthesis was severely impaired (*p* < 0.001, FDR < 1%), indicating that arginine biosynthesis is closely associated with cardiac function (Supplementary Figure 3A).

## Discussion

### Potential Markers of MI

This study identified a panel of discriminant metabolites that were also suggested as potential markers of MI in previous reports, such as taurine, methionine, leucine, isoleucine, valine, ornithine, tryptophan, citrate, and 2-ketoglutarate. Arginine biosynthesis and purine and pyrimidine metabolism pathways were also found to be seriously influenced in MI plasma samples. Among the differential metabolites between MI cases and non-MI cases, 10 of them in the MI group had more than twice the abundance as in the non-MI group (MIs/non-MIs, FC > 2); 17 metabolites had less than half the abundance as in the non-MI cases (MIs/non-MIs, FC < 0.5).

#### Elevated Markers in MI Cases

The pyrimidine metabolism pathway was also reprogrammed in MI cases. However, as an intermediate metabolite in pyrimidine metabolism, dU has never been suggested to play a role in cardiovascular diseases before. Previously, reports demonstrated how exogenous dU largely abolished the uptake of thymidine in bone marrow cells and how dU can also slow down the incorporation of deoxyguanidine and deoxyadenosine into DNA (22, 23). Moreover, elevation of dU has been identified as a potential adverse factor for nucleotide pool balance and mitochondrial function in the case of mitochondrial neurogastrointestinal encephalomyopathy (24, 25). Some studies reported the relations between dU accumulation and cancer progression (26). It is speculated that a high dU level may be a risk for MI patients because it may lead to problems about DNA incorporation. Consistent with a previous report suggesting the relationship of dU with insulin resistance (27), our correlation analysis also suggested that dU is partially affected by diabetes (correlation analysis vs. diabetes, *p* < 0.05, Pearson's *r* = 0.23), in addition to tobacco use and HDL-C level.

Accumulated metabolites in the MI plasma sample demonstrate abnormal methionine and cysteine metabolism. In this metabolic module, methionine is a precursor of homocysteine and homoserine is utilized in the biosynthesis of methionine. Homoserine was reported as a serum marker for cardiac disease in atherosclerosis patients with stent restenosis (28). A high level of methionine has been identified as atherogenic (29) and metabo-toxic (30). As a precursor of homocysteine, methionine elevation is supposed to be a negative signal for the development of CAD. A previous study showed that methionine in CAD cases is significantly higher than in controls and is a risk factor for CAD occurrence in unadjusted OR analysis (31). Our data further showed that methionine has the potential to be an independent biomarker for MI. Notably, we found that the diagnostic performance of biomarker candidates for MI varied with individual characteristics. When we studied individuals with a history of diabetes, methionine achieved an AUC score as high as 100% (sensitivity = 100.00%, specificity = 100.00%). For non-diabetic inpatients, the AUC score of methionine was only 73%, which is much lower than that of diabetic inpatients.

Our study suggests that a higher methionine, homoserine, or dU level has the potential in confirming MI diagnosis among chest pains and in contributing to the occurrence of MI. They could also be candidate predictors of future MI, but they still need to be further studied.

Moreover, methionine, homoserine, and dU also have the potential of being independent predictors of lipid plaque rupture. This is supported by the finding that logistic regression showed that the logOR^(ACS/Control)^ of dU is 40.767, methionine 10,596.739, and homoserine 434.394. After being adjusted for age, gender, diabetes/hypertension/smoking history, and lipid levels, the logOR^(ACS/Control)^ of methionine is 158,368.17, the logOR^(ACS/Control)^ of dU is 37.185, and that of homoserine is 628.728. The above data suggest that homoserine, dU, and methionine also have the potential to be indicators of ACS occurrence, which also has the potential to indicate coronary lipid plaque rupture. However, their ability to predict future MI or ACS occurrence needs further cohort studies.

#### Decreased Metabolites in MI Cases

Both high level and low level of risk markers in plasma were detected in the MI. Among all the discriminant metabolites that declined in MI cases, arginine was the most noteworthy (Figure 3C). Arginine ROC analysis showed an AUC = 0.8865 (MIs vs. non-MIs). Our study showed that as cardiac damage and function worsened (further elevation of cTnT, AST, CK-MB, and NT-proBNP levels), these plasma arginine biosynthesis-related metabolites dropped more (Table 3). Arginine is the primary source of a vasodilator—nitric oxide. The lack of plasma arginine hints a deficiency of vasodilator in MI patients. Some reported that diminished global arginine bioavailability is predictive of increased CAD risk (17).

Uracil, a pyrimidine found in RNA, was also significantly decreased in MI plasma. Figures 4D,F show the relationship of uracil and dU in a brief dU-centered pathway. The panel implies the demand for damage repair after a heart attack. This suggests that after MI occurs, the body continues to repair itself by producing more RNA or even DNA (32).

Elevation of the ATP by-product inosine has been detected in human plasma samples as early as 15 min after exercise-induced myocardial damage (7). However, the level of inosine dropped in MI cases in this study. It is possible that circulating inosine change after myocardial infarction is very time-sensitive: it rises immediately after the myocardial damage but dropped within 1–2 days.

### The Association Between Plasma Cells and Circulatory Metabolites

Although the causes of metabolite alterations could be many (e.g., gut microbial metabolites), we studied plasma transcriptomics data because circulating substances can cross the cell membranes and influence plasma metabolites most directly. We studied transcriptomics of circulating cells of MI patients and healthy controls from the GEO database (GSE48060). Differential genes between MI and controls were determined as those FDR < 0.05, FC > 1.2/FC < 0.8 (Supplementary Table 6). The metabolite–gene–disease interaction network analysis from MetaboAnalyst 3.0 (https://www.metaboanalyst.ca/MetaboAnalyst/Secure/network/MnetParamView.xhtml) showed the interaction network between differential metabolites from our study and differential genes from GSE48060 (Supplementary Figure 4A). Glucose, uric acid, cholesterol, glycine, and arginine are all important hubs with high-degree centrality.

Metabolite–gene interaction analysis showed that abnormal plasma level of purine metabolism intermediates ADP, ATP, GDP, and beta-alanine may relate to the expressions of plasma cells' *ADCY7, YES1, PTGDR*, etc. in mRNA level (Supplementary Figure 4B).

#### Plasma Cells Cannot Explain the Elevation of Plasma Methionine

Figure 4A shows methionine, homocysteine, and cystathionine in a panel. Inconsistent with the observed elevation of methionine, circulating transcriptomics of MI patients (GSE48060) showed that among the methionine abundance-related genes, methionine synthase *5-Methyltetrahydrofolate-Homocysteine Methyltransferase* (*MTR*) expression decreased and *Cystathionine* β*-synthase* (*CBS*, converting homocysteine to cystathionine) increased (*p* < 0.05, Figure 4B). The other genes involved in methionine turnover, including methionine-tRNA ligase (*MetRS*), *MrsB*/*MrsA* [*MARS*, reduced methionine-(S)-S-oxide), and *Mat2a*/*Mat1a (*methionine adenosyltransferase), remained statistically unchanged in plasma cells. The above data showed that plasma cells tend to utilize methionine and homocysteine to produce more cystathionine in MI blood samples. Considering that plasma cells cannot explain the elevation of plasma methionine, it is more likely that damaged cardiac tissues (or other tissues/germs) are responsible for that. A transcriptomics study of MI mouse model (induced by left anterior descending ligation, GEO accession: GSE775) revealed that in ischemic cardiac tissue, *MetRS* decreased significantly (*p* 0.05) in MI mouse cardiac samples, and the utilization of methionine is handicapped (Figure 4C).

#### dU Abundance Is Likely to Be Related to Blood Cells

Unlike methionine, the elevation of dU is likely to be related to injured cardiomyocytes or blood cells. Normally, little dU and purines can be detected in the plasma of a healthy volunteer. When there is pathological change of the tissue or the cells, intracellular substances are released and enter into the circulation system. According to the human metabolome database (HMDB0000012), dU is detected in the blood. However, we only identified dU peak in plasma, not in serum. According to GSE48060, cytidine deaminase (*CDA*) and uridine phosphorylase 1 (*UPP1*) are upregulated in MI-circulating cells (*p* < 0.05). *CDA* catalyzes the formation of deoxyuridine from deoxycytidine and *UPP1* catalyzes the reversible transformation of dU to uracil, and the above two upregulated enzymes can lead to dU elevation. Figure 4D shows the metabolites and metabolic enzymes in the dU-related pathway.

*CDA* and *UPP1* are both highly enriched in immune cells (mainly in neutrophils and monocytes). Immune cells increased in MI plasma samples. Figure 4G shows white blood cell (WBC) counts, neutrophil (NE) counts, monocyte (MO) and lymphocyte (LY) counts, and dU abundance in MI and non-MI cases. A previous study (GSE103182) showed that STEMI patients feature more neutrophils and *CDA* mRNA in plasma than NSTEMI patients. Consistent with this finding, our data showed that in the STEMI group, both dU and neutrophil counts were higher than those in the NSTEMI group (Supplementary Figure 3G).

On the one hand, dU originates from plasma neutrophils, and it could also originate from damaged and remodeling hearts. Our study on transverse aortic constriction (TAC) mouse models showed that cardiac *CDA* mRNA expression increased as *BNP* and *ANF* mRNA levels increased (Supplementary Figures 3D–F,H). It is possible that damaged human heart could also expressed more *CAD* and produce more dU. Hopefully, more plasma single-cell information (33, 34) and omics data (e.g., cfDNA methylome (35)) will reveal the mechanism behind the changes very soon.

### Limitations

As indicated by adjusted and unadjusted OR values, traditional risk factors, such as diabetes, hypertension, and smoking, had confounding effects on the candidate MI metabolite markers (36). In this study, diabetes and smoking also ranked as the marked risk factors for MI occurrence, but the history of hypertension was not (OR < 1, *p* > 0.05). As for the key substances in lipid metabolism, higher levels of TC and TG are risk factors for MI groups.

However, the OR of LDL-C scored 0.58 between cardiac chest pains and control, indicating that LDL-C is not a risk for healthy controls to develop into cardiac chest pains (Supplementary Table 6). Similarly, hypertension has been recognized as a risk for developing CAD, but it is not indicated as a risk factor in this study on MI. Considering that around 65% of inpatients had received medical treatment either with antilipidemic or antihypertensive drugs, or both, before blood collection, their HDL-C, LDL-C, and blood pressure levels may had been normalized or improved, so as to bring deviation of the data.

## Data Availability Statement

Metabolome data is freely available upon request via email (jiyea@cpu.edu.cn). Publicly available datasets were analyzed in this study. This data can be found online at the following links: https://www.ncbi.nlm.nih.gov/geo/query/acc.cgi?acc=GSE48060, https://www.ncbi.nlm.nih.gov/geo/query/acc.cgi?acc=GSE103182, https://www.ncbi.nlm.nih.gov/geo/query/acc.cgi?acc=GSE775, https://www.ncbi.nlm.nih.gov/geo/query/acc.cgi?acc=GSE59867.

## Ethics Statement

The studies involving human participants were reviewed and approved by Ethics Committee of the First Affiliated Hospital of Nanjing Medical University. The patients/participants provided their written informed consent to participate in this study.

## Author Contributions

XK and JA was responsible for the concept of the study. GW and JA provided the LC/MS and GC/MS platform. ZY, LW, and CL confirmed the diagnosis of MI and non-MI chest pain cases. NA, YL, and HT collected the blood samples, recorded the medical history of the volunteers, and prepared the plasma samples. NA, ZL, and RS performed the untargeted metabolomics. NA and MY analyzed the data. NA, MY, and JA produced the figures and tables. XK, NA, and YL wrote the manuscript. All authors contributed to the article and approved the submitted version.

## Conflict of Interest

The authors declare that the research was conducted in the absence of any commercial or financial relationships that could be construed as a potential conflict of interest.

## Abbreviations

MI: myocardial infarction
CAD: coronary artery disease
UA: unstable angina
AUC: area under the ROC curve
QC: quality control
IS: internal standard
VIP: variable importance
OR: odds ratio
dU: deoxyuridine
TAC: transverse aortic constriction
GEO: Gene Expression Omnibus
IDP: isopentenyl diphosphate
CDA: cytidine deaminase
UPP1: uridine phosphorylase 1.
