# Supplementary material for: Plasma Metabolites Alert Patients With Chest Pain to Occurrence of Myocardial Infarction
Source: Front Cardiovasc Med. 2021 Apr 23;8:652746. doi: 10.3389/fcvm.2021.652746 (PMC8103546; doi:10.3389/fcvm.2021.652746)
Supplement: Supplementary file 1 [file Table_1.DOCX]

**Supplementary Table**

**Table S1 The identified endogenous compounds in plasma using GC/MS system**

| Identification | Retention time (min) | Quant mass(m/z) |
| --- | --- | --- |
| Alanine | 5.56 | 116 |
| 2-Aminoadipate | 9.21 | 260 |
| 3-Aminoisobutyrate | 8.53 | 174 |
| Aminomalonate | 8.67 | 320 |
| Arachidonic acid | 12.88 | 217 |
| Asparagine | 9.92 | 231 |
| Aspartate-2TMS | 8.12 | 262 |
| Aspartate-3TMS | 8.70 | 232 |
| beta-Alanine | 8.36 | 248 |
| Cholesterol | 17.94 | 458 |
| Citrate | 10.77 | 273 |
| Creatinine | 9.26 | 329 |
| Cysteine | 9.20 | 220 |
| Cystine | 13.09 | 218 |
| 2,3-Dihydroxybutyrate | 7.85 | 292 |
| Fructose | 11.11 | 307 |
| Fumarate | 7.69 | 245 |
| Glucose-1 | 11.26 | 364 |
| Glucose-2 | 11.61 | 204 |
| Glutamate | 9.59 | 246 |
| Glutamine-3TMS | 10.50 | 156 |
| Glutamine-4TMS | 10.28 | 227 |
| Glycerate | 7.66 | 292 |
| Glycerol | 7.20 | 205 |
| [Glycerol 3-phosphate](https://hmdb.ca/metabolites/HMDB0000126) | 10.46 | 357 |
| Glycine-TMS | 5.73 | 102 |
| Glycine-2TMS | 7.48 | 248 |
| Glycolate | 5.26 | 205 |
| cis-9-Hexadecenoic acid | 11.74 | 311 |
| Histidine | 11.32 | 154 |
| Homocysteine | 8.24 | 234 |
| 2-Hydroxybutyrate | 5.83 | 131 |
| 2-Hydroxyisovalerate | 6.21 | 145 |
| 3-Hydroxybutyrate | 6.15 | 233 |
| Hydroxyproline | 8.65 | 158 |
| Indole-3-propanate | 12.14 | 202 |
| Isoleucine | 7.36 | 158 |
| alpha-Ketoglutarate | 9.32 | 198 |
| Lactate | 5.05 | 219 |
| Leucine | 7.17 | 158 |
| Linoleic acid | 12.65 | 337 |
| Lysine | 11.30 | 174 |
| Malate | 8.76 | 233 |
| Mannose | 11.29 | 174 |
| Methionine | 8.99 | 176 |
| Methyl myristate | 10.16 | 242 |
| Methylcysteine | 8.34 | 218 |
| Monomethylphosphate | 6.35 | 241 |
| 1-Monooleoylglycerol | 14.98 | 397 |
| 1-Monopalmitin | 14.21 | 371 |
| 1-Monostearin | 15.08 | 399 |
| Myo-inositol | 12.23 | 318 |
| Myristic acid | 10.83 | 285 |
| ^13^C_2_-Myristic acid | 10.84 | 287 |
| Nonanoic acid | 7.82 | 215 |
| Oleic acid | 12.68 | 339 |
| Ornithine-3TMS | 9.57 | 142 |
| Ornithine-2TMS | 10.39 | 174 |
| Oxalate | 5.85 | 190 |
| 2-Oxoisocaproiate/ketoleucine | 6.66 | 216 |
| Palmitic acid | 11.83 | 313 |
| Phenylalanine | 9.69 | 218 |
| Phosphate | 7.20 | 314 |
| Proline | 7.40 | 142 |
| Pyroglutamate | 9.03 | 156 |
| Pyrophosphoric acid | 9.92 | 451 |
| Pyruvate | 4.96 | 174 |
| Ribitol | 11.16 | 307 |
| Ribose | 10.92 | 217 |
| Salicylic acid | 8.95 | 267 |
| Serine | 7.88 | 218 |
| Stearic acid | 12.75 | 341 |
| Succinate | 7.48 | 247 |
| Threonate | 9.27 | 292 |
| Threonine | 8.09 | 218 |
| Thymine | 12.41 | 243 |
| alpha-Tocopherol | 17.66 | 502 |
| Tryptophan | 12.73 | 202 |
| Tyrosine | 11.39 | 218 |
| Urate | 12.20 | 441 |
| Urea | 6.84 | 204 |
| Valine-3TMS | 5.36 | 174 |
| Valine-2TMS | 6.70 | 218 |

TMS, trimethylsilylation.
