# Supplementary material for: Plasma Metabolites Alert Patients With Chest Pain to Occurrence of Myocardial Infarction
Source: Front Cardiovasc Med. 2021 Apr 23;8:652746. doi: 10.3389/fcvm.2021.652746 (PMC8103546; doi:10.3389/fcvm.2021.652746)
Supplement: Supplementary file 2 [file Table_2.DOCX]

**Supplementary Table**

**Table S2 The identified endogenous compounds in plasma using LC/MS system**

| Identification | Retention time (min) | Quant mass(m/z) |
| --- | --- | --- |
| N-Acetylornithine | 9.16 | 173.0826-173.1026 |
| Adenosine monophosphate | 8.72 | 346.0453-346.0653 |
| Adenosine triphosphate | 8.52 | 505.9779-505.9979 |
| ADP | 8.5 | 426.0116-426.0316 |
| Alanine | 9.18 | 88.0299-88.0499 |
| Allantoic acid | 8.64 | 175.0368-175.0568 |
| Allantoin | 8.02 | 157.0262-157.0462 |
| 2-Aminobenzoic acid | 5.61 | 136.0299-136.0499 |
| Asparagine | 9.28 | 131.0357-131.0557 |
| Biotin | 8.07 | 243.0704-243.0904 |
| Carbamoyl phosphate | 8.13 | 139.9649-139.9849 |
| Citrate | 8.82 | 191.0092-191.0292 |
| Citrulline | 9.3 | 174.0779-174.0979 |
| Cystathionine | 9.52 | 221.0496-221.0696 |
| Cytidine | 9.04 | 242.0677-242.0877 |
| Deoxyribose 1-phosphate | 2.48 | 213.0064-213.0264 |
| Deoxyuridine | 3.2 | 227.0568-227.0768 |
| Dihydroorotic acid | 3.03 | 157.0150-157.0350 |
| FAD | 8.6 | 784.1393-784.1593 |
| Fructose 1,6-bisphosphate | 8.77 | 338.9782-338.9982 |
| Beta-D-Fructose 6-phosphate | 8.77 | 259.0119-259.0319 |
| Fumarate | 8.69 | 114.9932-115.0132 |
| dGDP | 8.77 | 426.0116-426.0316 |
| Gluconic acid | 8.62 | 195.0405-195.0605 |
| Glucose 6-phosphate | 8.5 | 259.0119-259.0319 |
| Glutamic acid | 8.75 | 146.0354-146.0554 |
| Glutamine | 9.25 | 145.0513-145.0713 |
| 5-13C-Glutamine | 9.23 | 146.0551-146.0751 |
| Glutathione | 8.74 | 306.0660-306.0860 |
| Glycine | 9.2 | 74.0142-74.0342 |
| GSSG | 8.77 | 611.1342-611.1542 |
| dGTP | 8.79 | 505.9779-505.9979 |
| Guanosine monophosphate | 8.77 | 362.0402-362.0602 |
| Histidine | 9.8 | 154.0517-154.0717 |
| Homocysteine | 8.69 | 134.0176-134.0376 |
| Homoserine | 9.16 | 118.0404-118.0604 |
| 4-Hydroxybenzoic acid | 9.52 | 138.0217-138.0417 |
| Hypoxanthine | 8.21 | 135.0207-135.0407 |
| Inosine | 8.5 | 267.0630-267.0830 |
| Inosine 2'-phosphate | 8.74 | 347.0293-347.0493 |
| Isoleucine | 8.54 | 130.0768-130.0968 |
| 2-ketoglutarate | 8.35 | 145.0037-145.0237 |
| Lactate | 8.02 | 89.0139-89.0339 |
| Lysine | 14.91 | 145.0877-145.1077 |
| Malate | 8.67 | 133.0037-133.0237 |
| Methionine | 8.74 | 148.0332-148.0532 |
| Myo-Inositol | 9.55 | 179.0456-179.0656 |
| NAD | 8.61 | 663.0991-663.1191 |
| Nicotinic acid | 3.22 | 122.0142-122.0342 |
| Orotidine | 8.07 | 287.0416-287.0616 |
| Pantothenic acid | 8.07 | 218.0929-218.1129 |
| Phenylalanine | 8.45 | 164.0612-164.0812 |
| Phenylpyruvic acid | 8.8 | 163.0295-163.0495 |
| 2-Phosphoglyceric acid | 8.54 | 184.9751-184.9951 |
| 3-Phosphoglyceric acid | 8.84 | 184.9751-184.9951 |
| Proline | 8.92 | 114.0455-114.0655 |
| 2-Pyrocatechuic acid | 9.35 | 154.0166-154.0366 |
| D-Ribose 5-phosphate | 8.57 | 229.0014-229.0214 |
| D-Sedoheptulose 7-phosphate | 8.77 | 289.0225-289.0425 |
| Serine | 9.32 | 104.0248-104.0448 |
| Shikimic acid | 8.77 | 173.0350-173.0550 |
| Succinate | 8.23 | 117.0088-117.0288 |
| Taurine | 8.82 | 123.9969-124.0169 |
| Threonine | 8.8 | 118.0404-118.0604 |
| 5-Thymidylic acid | 8.57 | 321.0388-321.0588 |
| Trehalose | 9.28 | 341.0984-341.1184 |
| Tryptophan | 8.4 | 203.0721-203.0921 |
| Tyrosine | 8.71 | 180.0561-180.0761 |
| Ureidosuccinic acid | 3.74 | 175.0255-175.0455 |
| Uridine | 8.57 | 243.0517-243.0717 |
| Uridine 5'-diphosphate | 8.62 | 402.9844-403.0044 |
| Uridine 5'-monophosphate | 8.75 | 323.0181-323.0381 |
| Uridine diphosphate glucose | 8.72 | 565.0372-565.0572 |
| Valine | 8.86 | 116.0612-116.0812 |
| Xanthine | 8.02 | 151.0156-151.0356 |
| Xanthosine | 8.11 | 283.0579-283.0779 |
