# Supplementary material for: Plasma Metabolites Alert Patients With Chest Pain to Occurrence of Myocardial Infarction
Source: Front Cardiovasc Med. 2021 Apr 23;8:652746. doi: 10.3389/fcvm.2021.652746 (PMC8103546; doi:10.3389/fcvm.2021.652746)
Supplement: Supplementary file 3 [file Table_3.DOCX]

**Supplementary Table**

**Table S3 Pearson correlation analysis of homoserine, homocysteine, methionine and deoxyuridine to the traditional risk factors**

| Coefficients, r | TG | LDL-C | LPa | HDL-C | GLU | Gender | Hypertension | Diabetes | Smoke |
| --- | --- | --- | --- | --- | --- | --- | --- | --- | --- |
| Homoserine | 0.2 | 0.09 | 0.03 | -0.24* | 0.1 | 0.09 | 0.07 | 0.24* | 0.39* |
| Homocysteine | 0.08 | 0.08 | -0.01 | -0.12 | 0.02 | 0.11 | 0.09 | 0.11* | 0.24* |
| Methionine | 0.07 | 0.03 | 0.03 | -0.21* | 0.06 | 0.13 | 0.07 | 0.22* | 0.27* |
| Deoxyuridine | 0.11 | 0.02 | 0.01 | -0.27* | 0.07 | 0.08 | 0.09 | 0.23* | 0.24* |

*p<0.05, Pearson *r* correlation analysis
