# Supplementary material for: Plasma Metabolites Alert Patients With Chest Pain to Occurrence of Myocardial Infarction
Source: Front Cardiovasc Med. 2021 Apr 23;8:652746. doi: 10.3389/fcvm.2021.652746 (PMC8103546; doi:10.3389/fcvm.2021.652746)
Supplement: Supplementary file 4 [file Table_4.DOCX]

**Supplementary Table**

**Table S4 The metabolites differentiating NT-proBNP positive from negative (+/-) of the chest pain cases**

| Differential metabolites | NT-proBNP | |  |
| --- | --- | --- | --- |
|  | (+)(n=104) | (-)(n=29) | FC(+)/(-) |
| Uracil | 133379 | 245368 | 0.54 |
| alpha-Ketoglutarate | 35569 | 64328 | 0.55 |
| N-acetylornithine | 121902 | 207361 | 0.59 |
| Citrate | 2796824 | 4335952 | 0.65 |
| Xanthine | 133154 | 204184 | 0.65 |
| Cystathionine | 14009 | 21215 | 0.66 |
| Adenosine triphosphate | 31138 | 40746 | 0.76 |
| dGTP | 31138 | 40746 | 0.76 |
| Carbamoylphosphate | 26535 | 33950 | 0.78 |
| Glycine | 230409 | 174583 | 1.32 |
| Cystine | 16941 | 12464 | 1.36 |
| Aspartate | 11210 | 8199 | 1.37 |
| Threonine | 1210537 | 828762 | 1.46 |
| Homoserine | 1210537 | 828762 | 1.46 |
| Methionine | 276817 | 181468 | 1.53 |
| Deoxyuridine | 769653 | 300824 | 2.56 |

All the listed metabolites showed statistical difference by T test analysis between NT-proBNP positive and negative cases with their *logarized* data.

Metabolites with FC(+)/(-) from 0.8 to 1.2 were removed.
