# Supplementary material for: Plasma Metabolites Alert Patients With Chest Pain to Occurrence of Myocardial Infarction
Source: Front Cardiovasc Med. 2021 Apr 23;8:652746. doi: 10.3389/fcvm.2021.652746 (PMC8103546; doi:10.3389/fcvm.2021.652746)
Supplement: Supplementary file 5 [file Table_5.DOCX]

**Supplementary Table**

**Table S5 OR of traditional CAD risk factors**

| MI *vs* non-MI cases | | |
| --- | --- | --- |
|  | OR | 95% C.I. |
| TC | 1.80 | 1.1590-2.7998 |
| TG | 2.01 | 1.1039-3.6758 |
| LDL-C | 2.20 | 1.2381-3.8919 |
| Diabetes | 3.25 | 1.3632-7.7695 |
| Smoke | 2.48 | 1.2864-4.7674 |
| Cardiac chest pain cases *vs* the controls | | |
|  | OR | 95% C.I. |
| Age | 1.05 | 1.0291-1.0718 |
| TC | 0.54 | 0.3992-0.7195 |
| LDL-C | 0.58 | 0.4057-0.8211 |
| HDL-C | 0.00 | 0.0006-0.0176 |
| TP | 0.58 | 0.4907-0.6897 |
| ALB | 0.52 | 0.4289-0.6394 |
| A/G | 0.03 | 0.0070-0.1201 |
| Urea | 1.32 | 1.1062-1.5698 |
| Ca^2+^ | 0.00 | 0.0000-0.0000 |
