# Supplementary material for: Plasma Metabolites Alert Patients With Chest Pain to Occurrence of Myocardial Infarction
Source: Front Cardiovasc Med. 2021 Apr 23;8:652746. doi: 10.3389/fcvm.2021.652746 (PMC8103546; doi:10.3389/fcvm.2021.652746)
Supplement: Supplementary file 6 [file Table_6.DOCX]

**Supplementary Table**

**Table S6 Differential genes* between MI and healthy controls from GSE48060 database**

|  | **logFC** | **AveExpr** | **adj.P.Val** |
| --- | --- | --- | --- |
| RPS11 | 0.7019 | 9.7195 | 0.0013 |
| FOLR1 | 0.6400 | 8.7576 | 0.0084 |
| TMEFF2 | 0.5754 | 7.3664 | 0.0344 |
| LOC400499 | 0.5211 | 7.9406 | 0.0081 |
| SHKBP1 | 0.4127 | 7.5717 | 0.0344 |
| CNN2 | 0.3152 | 8.8741 | 0.0132 |
| UBA1 | 0.2720 | 6.9154 | 0.0404 |
| ADCY7 | -0.3267 | 9.6928 | 0.0318 |
| SDE2 | -0.3324 | 8.8539 | 0.0456 |
| AHSA2P | -0.3652 | 8.7476 | 0.0161 |
| UICLM | -0.4133 | 6.0580 | 0.0318 |
| CHST2 | -0.4212 | 8.0530 | 0.0456 |
| PTPRO | -0.4254 | 11.3160 | 0.0456 |
| INSIG1 | -0.4431 | 8.9796 | 0.0199 |
| GOLGA8N | -0.4442 | 10.4550 | 0.0114 |
| HEG1 | -0.4474 | 7.6692 | 0.0114 |
| TMEM70 | -0.4697 | 8.4421 | 0.0409 |
| TMEM64 | -0.4840 | 8.0479 | 0.0409 |
| PPM1L | -0.4851 | 6.3911 | 0.0269 |
| IL2RB | -0.5029 | 10.0453 | 0.0161 |
| RAB27B | -0.5153 | 9.2230 | 0.0456 |
| FASLG | -0.5223 | 5.7241 | 0.0133 |
| TBX21 | -0.5481 | 8.1790 | 0.0409 |
| YES1 | -0.5494 | 5.3671 | 0.0161 |
| SMAD7 | -0.5502 | 6.3136 | 0.0171 |
| ADAMTS1 | -0.5612 | 5.3072 | 0.0235 |
| GK5 | -0.5857 | 6.3427 | 0.0071 |
| PRF1 | -0.5943 | 10.6807 | 0.0344 |
| ENPP4 | -0.6157 | 7.0674 | 0.0409 |
| KLRD1 | -0.6850 | 8.9885 | 0.0314 |
| GZMB | -0.7216 | 9.7147 | 0.0151 |
| PTGDR | -0.7353 | 7.5064 | 0.0409 |
| AKR1C3 | -0.7428 | 6.9258 | 0.0344 |
| KLRB1 | -0.7729 | 10.2953 | 0.0071 |
| KLRF1 | -0.8230 | 9.7815 | 0.0114 |

*FDR 0.05, FC>1.2 or FC<0.8
